# The unintended impact of ecosystem preservation on greenhouse gas emissions: Evidence from environmental constraints on hydropower development in the United States

**DOI:** 10.1371/journal.pone.0210483

**Published:** 2019-01-10

**Authors:** Edson Severnini

**Affiliations:** Carnegie Mellon University (Heinz College) and IZA, Pittsburgh, PA, United States of America; University of California Irvine, UNITED STATES

## Abstract

Many countries have passed environmental laws aiming at preserving natural ecosystems, such as the Endangered Species Act of 1973 in the United States. Although those regulations seem to have improved preservation, they may have had unintended consequences in energy production. Here we show that while environmental constraints on hydropower may have preserved the wilderness and wildlife by restricting the development of hydroelectric projects, they led to more greenhouse gas emissions. Environmental regulations gave rise to a replacement of hydropower, which is a renewable, relatively low-emitting source of energy, with conventional fossil-fuel power, which is highly polluting. Our estimates indicate that, on average, each megawatt of fossil fuel power-generating capacity added to the grid because of environmental constraints on hydropower development led to an increase in annual carbon dioxide emissions of about 1,400 tons. Environmental regulations focusing only on the preservation of ecosystems appear to have encouraged electric utilities to substitute dirtier fuels for hydropower in electricity generation.

## Introduction

Environmental regulations have been long enacted in the United States and abroad to preserve the wilderness and wildlife. As a result, the natural habitat of many threatened/endangered species may have been protected [1–5]. Despite this invaluable benefit, such regulations might have an unintended consequence: more greenhouse gas (GHG) emissions. Indeed, environmental regulations may restrict the development of new hydroelectric dams, which are renewable, relatively low-emitting sources of energy, and might induce electricity generation by highly polluting firms: the conventional fossil-fuel power plants. As a matter of fact, conventional electricity generation is responsible for about 30 percent of U.S. GHG emissions, and is loosely regulated [6–7]. In 2017, 32 percent of the total electricity generated in the U.S. came from natural gas power plants, and 30 percent from coal power plants [7].

In this paper, I examine the trade-off between ecosystem preservation and GHG emissions. My empirical analysis is informed by a simple general equilibrium model for the electricity industry, presented in the methods section. I assume that consumers value electricity, ecosystem preservation and climate stability, but that electricity generation damages the environment, through either construction of hydroelectric dams or GHG emissions which contribute to climate change. Although evident under reasonable assumptions on the production and utility functions, the trade-off is not clear in the more general theoretical model. Therefore, it is an empirical question. As it will be explained in details below, my empirical strategy provides evidence pointing to the existence of that trade-off in the United States.

To proceed with the empirical analysis, I use two sources of data. The first is a unique report prepared in the 1990s for the U.S. Department of Energy (DOE) to determine the undeveloped potential hydropower resources in the U.S. It is the 1998 U.S. Hydropower Resource Assessment, prepared by the Idaho National Engineering and Environmental Laboratory (INL) [8–9]. It contains site characteristics such as exact location and potential generation capacity, and, crucially, the list of all land regulations that reduce the viability of each site, as well as the likelihood of development of each site, discounted by each regulation. Such information allows me to compute the hydropower potential that cannot be developed due to regulations meant to preserve the wilderness and wildlife, the focus of my analysis. The second source of data is “The Emissions & Generation Resource Integrated Database” [10], produced by EPA. It is a comprehensive database on the environmental characteristics for the vast majority of electric power produced in the U.S., including electricity generating capacity in power plants, and air emissions for carbon dioxide, sulfur dioxide, and nitrogen oxides, and from 2005 onwards, carbon dioxide equivalent (including methane and nitrous oxide).

To test empirically for the existence and economic relevance of the trade-off between ecosystem preservation and GHG emissions, I rely on an instrumental variable (IV) approach. My main outcome of interest is the county-level change in annual carbon dioxide emissions due to electricity generation over the period 1998–2014, which I relate to fossil-fuel electricity-generating capacity that was added to the power grid because of hydropower potential that could not be developed in that county due to regulations aiming at preserving the wilderness and wildlife. It is important to notice that although electric utilities do not have to balance electricity supply and demand at the county level, “[h]istorically, siting of electric power facilities has been regulated mainly on the state and local levels, *with local zoning commissions having the greatest influence*” ([11], p. xxv, my emphasis). The “Not In My Back Yard” (NIMBY) activism may have also made power plant site selection a local issue. Indeed, NIMBY activism has blocked proposed power plants by organizing local opposition, changing zoning laws, opposing permits, filing lawsuits, and using other long delay mechanisms [12]. In addition, the trade-off between electricity-generating technologies does seem to be salient at the local level. In fact, there has been an effort to systematize the comparison across electricity-generating technologies assuming the county as the unit of analysis [13].

Using instrumental variable estimation, I uncover the local average treatment effect (LATE–[14]) of changes in fossil-fuel electricity-generating capacity associated with hydropower not developed because of environmental constraints. The main finding is that each megawatt of fossil fuel capacity installed because of ecosystem preservation regulations constraining hydropower development leads to an increase in annual carbon dioxide emissions of about 1,400 tons. To emphasize the LATE interpretation of this estimate, this is the average treatment effect for the counties whose fossil-fuel electricity-generating capacity was influenced by constraints on hydropower development. To put that estimate in perspective, I compare it with the average annual emissions per megawatt of the 2016 U.S. power plant fleet– 1,795 tons (calculations based on data available at eia.gov/electricity/state/unitedstates/). This similarity suggests that the trade-off between ecosystem preservation and GHG emissions does seem to manifest at least in part at the local level.

This paper makes two contributions to the literature and the design of environmental policies in the U.S. and abroad. First, it highlights the pernicious incentives that incomplete environmental regulations generate, and points to the importance of an integrated regulatory framework which includes both ecosystem preservation and GHG emissions (see [15] for related issues regarding incomplete environment regulation). If the government seeks to preserve nature via regulations, it may have to restrict land use and emissions at the same time. (The cost, however, might be higher relative prices for electricity, as predicted by my simplified model.) Such a regulatory framework may be useful to guide the debate on the development of other renewable energy sources, such as wind and solar energy. Studies have identified enough sites in the U.S. that, if developed, could make these technologies the dominant electricity sources in the country [16]. Nevertheless, large-scale wind and solar projects might create major alterations to the landscape and would not be seen as environmentally friendly, as in the hydroelectricity case. Indeed, wind turbines may harm many species of birds and bats, and large-scale solar projects in the desert may endanger the habitat of native animals [17–20]. Therefore, the trade-off between ecosystem preservation and emissions might be as fundamental as in the hydroelectricity context.

Second, while a few studies have investigated the consequences of restricting the operation of hydroelectric dams decades after construction due to environmental laws–the intensive margin response–this study examines the trade-off between ecosystem preservation regulations and GHG emissions arising from the construction of fossil fuel power plants instead of hydropower plants–the extensive margin response. The extensive margin should be also taken into account in power plant siting and permitting decisions by the U.S. Federal Energy Regulatory Commission (FERC). Regarding the previous studies, models have been used to estimate the cost of environmental constraints on hydropower operations on the Colorado River [21], Green River [22], and Snake River [23]. Flow constraints on the Manistee River are shown to lead to increases in thermal power generation during peak periods, and decreases during off-peak periods [24]. Previous research has found that replacement power generated from fossil fuels can increase or decrease emissions relative to baseline hydropower operations. With flow constraints on the Colorado River, the need for thermal power follows the pattern on the Manistee River, i.e., increasing during peak-demand periods and decreasing during off-peak periods [21], [24]. Thus, the net effect is a decrease in air pollution and GHG emissions [25]. In contrast, in the Columbia River basin, estimated air pollution and GHG emissions would increase in response to most environmental constraints on hydropower operations [26]. There, the time pattern of hydroelectricity production is not the primary adjustment. Because of those constraints, turbines were to be removed from service or hydraulic head severely reduced.

The remainder of the paper is organized as follows. The Materials and Methods section (i) presents a simple conceptual framework to illustrate the trade-off between ecosystem conservation and GHG emissions, (ii) provides more information about the databases used in this study, (iii) describes the hydropower assessment data and the suitability for development index, and (iv) outlines the methodology for the empirical analysis. The Results and Discussion section reports and discusses results. Lastly, the Conclusion section provides some concluding remarks.

## Materials and methods

### Conceptual framework

To examine the trade-off between ecosystem preservation and GHG emissions, I set up a simple general equilibrium model for electricity generation. Assume that consumers value electricity, ecosystem preservation and climate stability, but that electricity generation damages the environment, through either construction of hydroelectric dams or GHG emissions which contribute to climate change.

#### Set-up

In the simplest-possible setting, suppose there are two price-taking economic agents, a single consumer and a single firm, and three goods, land, climate stability, and electricity produced by the firm.

The consumer has a strictly concave utility function *U(T*,*C*,*A)*, defined over his consumption of electricity *T*, land for preservation *C*, and climate stability *A*. She has an endowment of *L* units of land and an endowment of *E* units of emission permits, but no endowment of electricity.

The firm uses inputs land *L* (to construct hydroelectric dams) and GHG emissions *E* to produce electricity according to the increasing and strictly concave production function *F(L*,*E)*. Thus, to produce the output, the firm must buy land and emission permits from the consumer. Assume that the firm seeks to maximize its profits, taking market prices as given. Letting *p*_*T*_ be the price of its output, *p*_*L*_ be the price of land, and *p*_*E*_ the price of emission permits, the firm solves

$$Max_{(L,E)\in R_{+}^{2}}\;p_{T}F(L,E)-p_{L}L-p_{E}E.$$ (1)

Given prices (*p*_*T*_, *p*_*L*_, *p*_*E*_), the firm’s optimal demands are *L*(*p*_*T*_, *p*_*L*_, *p*_*E*_) and *E*(*p*_*T*_, *p*_*L*_, *p*_*E*_), its output is *Q*(*p*_*T*_, *p*_*L*_, *p*_*E*_), and its profits are *π*(*p*_*T*_, *p*_*L*_, *p*_*E*_).

Firms are owned by consumers. Thus, assume that the consumer is the sole owner of the firm and receives the profits earned by the firm *π*(*p*_*T*_, *p*_*L*_, *p*_*E*_). Therefore, the consumer’s problem, given prices (*p*_*T*_, *p*_*L*_, *p*_*E*_), is

$$Max_{(T,L,E)\in R_{+}^{3}}\;U(T,C,A)$$ (2)

$$s.t.\;p_{T}T\leq p_{L}(\bar{L}-C)+p_{E}(\bar{E}-A)+\pi (p_{T},p_{L},p_{E}).$$

The budget constraint in (2) reflects the three sources of the consumer’s purchasing power. If the consumer supplies ($\bar{L}-C$) units of land for the construction of hydroelectric dams, and ($\bar{E}-A$) units of emission permits when prices are (*p*_*T*_, *p*_*L*_, *p*_*E*_), then the total amount she can spend on electricity is ($p_{L}(\bar{L}-C)$) + $p_{E}(\bar{E}-A)$ plus the profit distribution from the firm *π*(*p*_*T*_, *p*_*L*_, *p*_*E*_). The consumer’s optimal levels of demand in problem (2) for prices (*p*_*T*_, *p*_*L*_, *p*_*E*_) are denoted by (*T*(*p*_*T*_, *p*_*L*_, *p*_*E*_), *C*(*p*_*T*_, *p*_*L*_, *p*_*E*_), *A*(*p*_*T*_, *p*_*L*_, *p*_*E*_)).

A Walrasian equilibrium in this economy involves a price vector (p^*^_T_, p^*^_L_, p^*^_E_) at which the electricity, the land and the permit markets clear; that is, at which

$$Q(p_{T}^{*},p_{L}^{*},p_{E}^{*})=T(p_{T}^{*},p_{L}^{*},p_{E}^{*}),$$

$$L(p_{T}^{*},p_{L}^{*},p_{E}^{*})=\bar{L}-C(p_{T}^{*},p_{L}^{*},p_{E}^{*}),$$

$$E(p_{T}^{*},p_{L}^{*},p_{E}^{*})=\bar{E}-A(p_{T}^{*},p_{L}^{*},p_{E}^{*}).$$

As is well-known [27], *a particular electricity-land-permit combination can arise in a competitive equilibrium if and only if it maximizes the consumer’s utility subject to the economy’s technological and endowment constraints*. Indeed, the Walrasian equilibrium allocation is the same allocation that would be obtained if a planner ran the economy to maximize the consumer’s well-being. Therefore, the competitive equilibrium is also Pareto optimal. The equilibrium problem is

$$Max_{(T,L,E)\in R_{+}^{3}}\;U(T,C,A)$$

s.t.T=F(L,E),

$$C=\bar{L}-L,$$

$$A=\bar{E}-E,$$

which is equivalent to

$$Max_{(L,E)\in R_{+}^{2}}\;U(F(L,E),(\bar{L}-L),(\bar{E}-E)).$$ (3)

The first-order conditions for this problem are

$$U_{T}F_{L}-U_{C}=0,$$

$$U_{T}F_{E}-U_{A}=0,$$

from which we can define $L^{*}(\bar{L},\bar{E})$ and $E^{*}(\bar{L},\bar{E})$. To check for a trade-off between optimal emissions (E*) and land regulation (change in $\bar{L}$), we use the implicit function theorem and find

$$\frac{\partial E^{*}}{\partial \bar{L}}=\frac{\left(U_{T}U_{TC}F_{L}F_{LE}-U_{T}U_{TC}F_{E}F_{LL}+U_{T}U_{CC}F_{LE}+U_{T}U_{CA}F_{LL}\right)}{\begin{array}{c} (-U_{T}U_{TA}F_{L}F_{LE}-U_{TA}U_{CA}F_{L}-U_{T}U_{TC}F_{E}F_{LE}+U_{T}^{2}F_{LE}^{2}+U_{T}U_{CA}F_{LE}-U_{TC}U_{CA}F_{E}\\ +U_{T}U_{CA}F_{LE}+U_{CA}^{2}+U_{T}U_{TC}F_{L}F_{EE}-U_{TC}U_{AA}F_{L}+U_{T}U_{TA}F_{E}F_{LL}-U_{T}^{2}F_{EE}F_{LL}\\ +U_{T}U_{AA}F_{LL}-U_{TA}U_{CC}F_{E}+U_{T}U_{CC}F_{EE}-U_{CC}U_{AA}). \end{array}}$$

As we can see, the sign of $\partial E^{*}/\partial \bar{L}$ is ambiguous under mild assumptions for both the utility and the production functions, so we now turn to some special cases.

#### Case 1: Cobb-Douglas production and Cobb-Douglas utility

To illustrate the trade-off between ecosystem preservation and climate stability, let us find the competitive equilibrium for electricity generation using a Cobb-Douglas functional form for both the production function and the utility function. Let us obtain the equilibrium allocations (T^*^, L^*^, E^*^) first, and then compute the equilibrium prices (p^*^_T_, p^*^_L_, p^*^_E_). The competitive equilibrium problem is

$$Max_{(T,L,E)\in R_{+}^{3}}\;T^{\alpha }C^{\beta }A^{\gamma },\;\{\alpha ,\beta ,\gamma \}\in (0,1),$$

$$s.t.\;T=L^{l}E^{e},\;\{l,e\}\in (0,1),$$

$$C=\bar{L}-L,$$

$$A=\bar{E}-E,$$

which is equivalent to

$$Max_{(L,E)\in R_{+}^{2}}\;{\left(L^{l}E^{e}\right)}^{\alpha }{\left(\bar{L}-L\right)}^{\beta }{\left(\bar{E}-E\right)}^{\gamma }.$$ (4)

Solving problem (4) yields optimal allocations

$$L^{*}=\left(\frac{1}{1+(\frac{\beta }{\alpha l})}\right)\bar{L}\in (0,\bar{L}),$$ (5)

$$E^{*}=\left(\frac{1}{1+(\frac{\gamma }{\alpha e})}\right)\bar{E}\in (0,\bar{E}),$$ (6)

$$T^{*}={\left(L^{*}\right)}^{l}{\left(E^{*}\right)}^{e}.$$ (7)

Now, let (p^*^_T_, p^*^_L_, p^*^_E_) be a supporting price vector of the Pareto optimal allocations just identified. As a normalization, put p^*^_T_ = 1. The zero-profit condition and the cost minimization condition of the electricity production imply that

$$p_{L}^{*}=\frac{{\left(E^{*}\right)}^{e}}{{\left(1+\frac{e}{l})(L^{*}\right)}^{1-l}},$$ (8)

$$p_{E}^{*}=\frac{e}{l}\frac{{\left(L^{*}\right)}^{l}}{{\left(1+\frac{e}{l})(E^{*}\right)}^{1-e}},$$ (9)

$$\frac{p_{E}^{*}}{p_{L}^{*}}=\frac{e}{l}\frac{L^{*}}{E^{*}}.$$ (10)

To make the trade-off between ecosystem preservation and GHG emissions clear, let us write E^*^ as a function of L^*^ (and, consequently, $\bar{L}$). Hence,

$$E^{*}=\left(\frac{1}{1+\frac{\gamma }{\frac{e\beta }{l}(\frac{L^{*}}{\left(\bar{L}-L^{*}\right)})}}\right)\bar{E}.$$ (11)

Now, it is straightforward to see that optimal emissions E^*^ increase when governmental regulations restrict the number of developable sites for hydroelectric dams, that is, when government reduces $\bar{L}$. *Therefore*, *nature is being damaged one way or another*: *preservation of land is being offset with emission of greenhouse gases*. (Notice that the relative price of emissions with respect to land, p^*^_E_ / p^*^_L_, goes down with the ecosystem regulations. This is how the market accommodates the presence of such regulations.)

If the government does aim at implementing an eco-friendly policy, it should impose both ecosystem and emission regulations, that is, it should bring both $\bar{L}$ and $\bar{E}$ down. Indeed, Eq (11) says that the increase in optimal emissions arising from a lower $\bar{L}$ might be offset with a reduction in $\bar{E}$. The consequence of such a policy, however, is less electricity generation (see Eqs (5), (6) and (7)). As a result, the relative price of electricity might go up (see Eqs (8) and (9) and recall the normalization p^*^_T_ = 1). Ecosystem regulations have existed for a long time in the U.S. but emissions of greenhouse gases have yet to be regulated. The EPA is currently taking steps towards repealing the so-called “Clean Power Plan”, a policy aiming at controlling GHG emissions from power plants.

#### Case 2: Perfect substitutes production and Cobb-Douglas utility

The competitive equilibrium problem in this case is

$$Max_{(T,L,E)\in R_{+}^{3}}\;T^{\alpha }C^{\beta }A^{\gamma },\;\{\alpha ,\beta ,\gamma \}\in (0,1),$$

$$s.t.\;T=lL+eE,\;\{l,e\}\in R_{+},$$

$$C=\bar{L}-L,$$

$$A=\bar{E}-E,$$

which is equivalent to

$$Max_{(L,E)\in R_{+}^{2}}\;{\left(lL+eE\right)}^{\alpha }{\left(\bar{L}-L\right)}^{\beta }{\left(\bar{E}-E\right)}^{\gamma }.$$ (12)

Solving problem (12) yields optimal allocations

$$L^{*}=\frac{(\alpha +\gamma )l\bar{L}-\beta e\bar{E}}{(\alpha +\beta +\gamma )l}\in (0,\bar{L}),$$ (13)

$$E^{*}=\frac{(\alpha +\beta )e\bar{E}-\gamma l\bar{L}}{(\alpha +\beta +\gamma )e}\in (0,\bar{E}),$$ (14)

$$T^{*}=lL^{*}+eE^{*}.$$ (15)

Now, let (p^*^_T_, p^*^_L_, p^*^_E_) be a supporting price vector of the Pareto optimal allocations just identified. As a normalization, put p^*^_T_ = 1. Also, due to the perfect substitutability of inputs in the production function, and the non-zero optimal allocations for both of them, p^*^_E_ = (e/l) p^*^_L_. The zero-profit condition of the electricity generation then implies that

$$p_{L}^{*}=l,$$ (16)

$$p_{E}^{*}=e,$$ (17)

Notice that the trade-off between ecosystem preservation and GHG emissions is again evident in Eq (14).

### Data sources

As explained briefly in the introduction, to proceed with the empirical analysis, I use two sources of data. The first is a unique report prepared in the 1990s for the U.S. Department of Energy (DOE) to determine the undeveloped potential hydropower resources in the U.S. It is the 1998 U.S. Hydropower Resource Assessment, prepared by the Idaho National Engineering and Environmental Laboratory (INL) [8–9]. It contains site characteristics such as exact location and potential generation capacity, and, crucially, the list of all land regulations that reduce the viability of each site, as well as the probability of development of each site, discounted by each regulation. Such information allows me to compute the hydropower potential that cannot be developed due to regulations meant to preserve the wilderness and wildlife, the focus of my analysis. The additional regulations are used for a falsification test, which I explain later in the results section.

The second source of data is “The Emissions & Generation Resource Integrated Database” [10], produced by EPA. It is a comprehensive database on the environmental characteristics for the vast majority of electric power produced in the U.S., including electricity generating capacity in power plants, and air emissions for carbon dioxide, sulfur dioxide, and nitrogen oxides, and from 2005 onwards, carbon dioxide equivalent (including methane and nitrous oxide). Most of the eGRID information, including plant opening years, comes from the U.S. Department of Energy’s Annual Electric Generator Report compiled from responses to the EIA-860, a form completed annually by all electric generating plants. In addition, eGRID includes plant identification information, geographic coordinates, primary fuel, number of generators, and plant annual net generation.

### Hydropower assessment and suitability for development

The INL report–crucial source of information for my analysis–presents DOE’s efforts to produce a more definitive assessment of undeveloped hydropower resources within the U.S. No agency had previously estimated the undeveloped hydropower capacity using such a comprehensive database including site characteristics, stream flow data, and available hydraulic heads. Initial efforts began in 1989 and information from the last state was received in 1998. State agencies such as departments of dam safety, water resources, environmental quality, fish and game, history, and commerce, contributed information about hydropower resources within their states. The report summarizes and discusses the undeveloped conventional hydropower capacity for the 5,677 sites within the country. It does not include the capacity produced by pumped storage sites. However, for conventional hydropower, the resource assessment contains site identification information, geographic coordinates, and crucially, the estimated nameplate capacity–the intended technical full-load sustained output of a facility.

#### Suitability factor determination and undevelopable hydropower potential

A key element of my analysis is the suitability factor of a potential hydropower site. This factor reflects the probability that environmental considerations might make a project site unacceptable, prohibiting its development. Suitability factors were developed by the INL, in conjunction with Oak Ridge National Laboratory staff who are experienced in hydropower licensing cases. Five potential values were selected, as shown in Table 1 (Panel A1). (The discussion that follows is heavily based on [8].)

**Table 1:** Valuation of environmental attributes in the hydropower assessment.

**Panel A1. Effect of Environmental Attribute**	**Value of Suitability Factor**
Least impediment to development	0.90
Minor reduction in likelihood of development	0.75
Likelihood of development reduced by half	0.50
Major reduction in likelihood of development	0.25
Development prohibited or highly unlikely	0.10
**Panel A2. Environmental Attributes and Suitability Factors**	**Overall Project Suitability Factor**
No environmental attributes assigned	0.90
Lowest individual factor(s) = 0.90	0.90
Lowest individual factor = 0.75	0.75
Two or more lowest individual factors = 0.75	0.50
Lowest individual factor = 0.50	0.50
Two or more lowest individual factors = 0.50	0.25
Lowest individual factor = 0.25	0.25
Two or more lowest individual factors = 0.25	0.10
Lowest individual factor(s) = 0.10	0.10

*Source*: Conner, Francfort, and Rinehart (1998, p.11-13).

The crucial step in evaluating the environmental suitability of each project site is to combine the suitability factors for the individual environmental attributes into a single factor for each project site. This overall suitability factor is an estimate of the probability of a project’s successful development, considering all the attributes described below. The presence of more than one environmental attribute means that more than one environmental concern affects a project. The overall suitability factor should be no greater than the lowest factor for individual attributes, and it should be less than the lowest factor if multiple significant environmental constraints are present. For example, if an undeveloped project has both fish values (suitability factor = 0.25) and wildlife values (suitability factor = 0.25), the cumulative effects of these two concerns will make its overall suitability even less than 0.25, so an overall suitability factor of 0.1 is assigned.

If the environmental suitability factors for individual attributes were truly the probability of the project’s being developed, then the overall probability of development could be mathematically calculated. And, if the individual suitability factors were true and independent probabilities, then the probability of developing the project site because of environmental concerns would be equal to the product of all the individual factors. However, the FERC’s licensing process is not a statistical probability function, and it cannot be assumed that suitability factors can be handled as independent probabilities (for example, there is a strong correlation between the scenic, recreational, and fishing values of a stream). When FERC issues a license mandating a certain level of environmental protection, it is implicitly choosing a certain trade-off between environmental protection and hydropower production. Nevertheless, other factors such as legislative, social, and institutional constraints, as described below, and collaborative governance also affect FERC’s regulatory decisions [12], [28–33]. The procedure outlined in Table 1 (Panel A2) is used for assigning overall suitability factors. It was developed by the laboratories mentioned above and assumes that the lowest suitability factor dominates the likelihood of a project’s development. However, it also considers the reduced likelihood of development resulting from the occurrence of multiple low suitability factors.

After finding the overall suitability factor for each potential hydropower site, I obtain the likelihood of development at the county level. First, I weight the potential capacity of each site with its own likelihood, and sum the weighted capacities over all sites in the county. Then, I divide this weighted sum by the total potential capacity. This quotient is my county suitability factor.

To obtain a crucial variable in my analysis–the hydropower potential that cannot be developed because of environmental constraints in each county–I use the likelihood that some hydroelectric projects will not be carried out, which is one minus the county suitability factor. This likelihood of non-development is then multiplied by the hydropower potential at the county level.

#### Environmental, legal, and institutional attributes

The INL defined the following environmental, legal, and institutional attributes. I use only the ones marked with (*) as the environmental constraints on hydropower development in my empirical analysis. The corresponding suitability factors are fully explained in the subsection above.

Wild/Scenic Protection (*). This attribute identifies project sites that are included in the federal wild and scenic rivers system, under consideration for inclusion in the federal system, included in a state river protection program, in a designated wilderness area, or protected from development under another program. Relatively few sites have this status, but those that do are highly unlikely to be developed. Projects at undeveloped sites on state or federally protected wild and scenic rivers, or in wilderness areas, must be assumed to be legally protected from hydropower development. Also, projects at sites under consideration for protection are highly likely to be opposed by state and federal resource agencies, and protection will be approved at many such sites before hydropower development could occur. Because it is possible, but highly unlikely, that development could occur at a site with wild and scenic river protection, the suitability factor assigned to all such projects at undeveloped sites is 0.1. It is highly unlikely that a project at an existing dam would be on a wild and scenic river since rivers are usually designated as wild and scenic only if they are free of developments such as dams. A suitability factor of 0.5 is assigned for such unusual cases.

Wild and Scenic Tributary or Upstream or Downstream of a Wild and Scenic Location (*). This attribute is assigned to a project if it is at the upstream or downstream end of a wild and scenic river reach or is on a tributary of a wild and scenic river. A project at a developed site would affect a downstream wild and scenic river if additional alterations to the flow regime resulted. A suitability factor of 0.75 is assigned for such projects. Projects at undeveloped sites are highly likely to alter the flow regime and may cause changes in downstream water quality, so a suitability factor of 0.5 is assigned to undeveloped sites.

Cultural and Historic Values. Project impacts on cultural and historic resources can often be mitigated (for example, by excavating archeological sites or relocating historic structures). Projects at existing dams are unlikely to affect such resources unless an increase in reservoir pool elevation occurs or major new structures are built. A suitability factor of 0.75 is assigned to such projects. Development of undeveloped sites is more likely to affect cultural and historic resources, so a suitability factor of 0.5 is assigned.

Fish Presence Value (*). A stream reach may or may not have legally protected fisheries. In either case, however, strong state opposition to new development must be expected if a valuable fishery resource exists. Relatively high instream flow release requirements can mitigate the impact on fisheries, but a high instream flow release would reduce the economic viability of the project. Projects at developed sites could have some impact, such as increased turbine mortality. A suitability factor of 0.75 is assigned to projects at developed sites. Development at undeveloped sites could have a major impact on aquatic habitat through inundation, migration blockage, turbine mortality, water quality, and altered flows. Some of these can be mitigated, but such mitigation could be expensive. A suitability factor of 0.25 is assigned to undeveloped sites.

Geologic Value. Geologic values such as rock formations are rarely protected legally and are not generally affected by small projects. Development at existing sites is not affected by geologic resources, so a suitability factor of 0.9 is assigned. Development at undeveloped sites may inundate geologic features, so a suitability factor of 0.5 is assigned.

Recreation Value. River recreation users tend to be effective opponents of hydropower development. Development at any storage dam would affect recreation by altering flow releases; mitigation typically includes higher flow releases during periods of high recreation use. Such releases can be made through turbines, but higher flow releases tend to occur when power demands are low. Projects at existing dams would have little effect on recreation besides flow alterations, so they are assigned a suitability factor of 0.75. Projects at undeveloped sites would inundate reaches, block the passage of boats, and reduce aesthetics. Because projects at undeveloped sites are likely to be strongly opposed, a suitability factor of 0.25 is assigned.

Scenic Value. Scenic values are not legally protected but must be considered in assessing the impact of a project. Scenic values are also important to recreational river users. The addition of power to existing dams would alter scenic values only through the addition of new structures and perhaps by reducing visually attractive spillage, so a suitability factor of 0.9 is assigned. New projects at undeveloped sites would have important effects on scenic resources because views would be altered by the project. Undeveloped projects are assigned a suitability factor of 0.5.

Wildlife Value (*). Terrestrial wildlife and wildlife habit are protected by fish and game agencies that are influential in determining mitigation requirements for hydropower projects. Development at existing sites would have little effect on wildlife unless reservoir pool elevations were altered or construction of major facilities was required. A suitability factor of 0.75 is assigned for projects at existing sites. Development at undeveloped sites could inundate wildlife habitat, and construction would cause a great deal of disturbance. It is difficult to mitigate such impacts, so opposition to such a project could be strong. Undeveloped projects are assigned a suitability factor of 0.25.

Other Values. The effects of other values, such as the presence of rare wetland communities or consideration for wilderness designation, are assigned by using the most commonly assigned suitability factor for the other values. For projects at developed sites, the suitability factor is 0.75. For projects at undeveloped sites, the suitability factor is 0.5.

Threatened and Endangered Fish or Wildlife (*). The presence of threatened and endangered species near a project site requires additional consultations with wildlife agencies and can result in additional studies and mitigation requirements. The presence of threatened and endangered fish species may preclude development of new storage projects because new projects can involve the greatest alteration of aquatic habitat. Terrestrial threatened and endangered species are unlikely to be highly affected by run-rivers projects, but storage reservoirs could affect terrestrial habitat. For existing sites, a suitability factor of 0.75 is assigned when threatened and endangered species are present. For projects at undeveloped sites, a suitability factor of 0.5 is assigned when threatened and endangered species are present.

Federal Land Code 103: National Park, Monument, Lakeshore, Parkway, Battlefield, or Recreation Area. These lands are legally protected from development. A suitability factor of 0.1 is assigned for such projects.

Federal Land Code 104 (*): National Forest or Grassland. These lands are not legally protected from development, but the managing agency has the right to impose additional mitigation requirements on projects. A suitability factor of 0.75 is assigned to projects at existing sites, since these projects typically have fewer impacts. A suitability factor of 0.5 is assigned for undeveloped sites.

Federal Land Code 105 (*): National Wildlife Refuge, Game Preserve, or Fish Hatchery. These lands are managed for fish and wildlife habitats, and hydropower development would almost always be incompatible. A suitability factor of 0.1 is assigned for such projects.

Federal Land Code 106 (*): National Scenic Waterway or Wilderness Area. These lands are legally protected from development. A suitability factor of 0.1 is as- signed for such projects.

Federal Land Code 107: Indian Reservation. These lands are not legally protected from development, but Indian tribes have the right to impose additional mitigation requirements on projects. A suitability factor of 0.75 is assigned for projects at developed sites, and a suitability factor of 0.5 is assigned for projects at undeveloped sites.

Federal Land Code 108: Military Reservation. These lands are not legally protected from development, but the managing agency has the right to impose additional mitigation requirements on projects. A suitability factor of 0.75 is assigned for projects at developed sites, and a suitability factor of 0.5 is assigned for projects at undeveloped sites.

Federal Land Code 198: Not on Federal Land. This variable indicates that the project is not on federal land, so there are no development constraints based on federal land codes. The value for this variable is 0.9.

### Empirical strategy

In order to test empirically for the existence and economic relevance of the trade-off between ecosystem preservation and GHG emissions, I use ideas advanced in the conceptual framework developed previously. Basically, I regress the change in carbon dioxide emissions (Δ*E*) in county (*c*) over the period 1998–2014 on fossil-fuel electricity-generating capacity added to the power grid during the same period of time (Δ*FossFuelCap*), controlling for changes in total employment (Δ*Emp*) and per capita income (Δ*PCInc*), and a set of Census Division (*d*) fixed effects (*η*),

$$\Delta E_{c}=\beta_{0}+\beta_{1}\Delta FossFuelCap_{c}+\beta_{2}\Delta Emp_{c}+\beta_{3}\Delta PCInc_{c}+\eta_{d}+\varepsilon_{c},$$ (18)

The coefficient of interest in Eq (18) is *β*_*1*_. It reflects the average effect of a 1-megawatt increase in thermal power capacity on carbon dioxide emissions. We cannot interpret this coefficient as a causal effect because changes in important unobserved variables such as preference for GHG emissions and the preservation of the wilderness and wildlife, and access to coal and natural gas at a local level, may be correlated with the choice of technology used for electricity generation. Because county fixed effects are included in the analysis (they are netted out in the differencing strategy), local fossil fuel reserves are controlled for in the estimation. Similarly, because Census Division fixed effects are included in the differenced estimating equation (implicitly controlling for differential trends based on Census Divisions), preferences and natural resources exploration are allowed to vary over time across the nine U.S. Census Divisions. Notwithstanding, it is still possible to have omitted variable bias because of varying preferences and availability of fossil fuels at the county level over time. The bias could be positive or negative. Because preferences for environmental amenities might be negatively related to investments in fossil fuel electricity generating capacity, and also negatively related to GHG emissions, then the bias may be positive. On the other hand, emission levels are likely to be higher around metropolitan areas where economic activity and more-educated, amenity-oriented individuals are more abundant [34–35]. Hence, the bias could be negative.

To address that endogeneity issue, I use an instrumental variable (IV) strategy to identify the causal response of GHG emissions to changes in thermal power capacity. My instrument is the potential hydropower capacity that cannot be developed due to environmental constraints in county *c* (*HydroNotDev*_*c*_). Because *HydroNotDev* represents the decrease in land availability for the development of new hydro dams ($\bar{L}$ in the theoretical model) due to environmental constraints, the IV estimate for *β*_*1*_ captures the increase in emissions arising from the potential substitution of hydropower plants with fossil-fuel power plants in electricity generation. Therefore, it reveals the relevance of the aforementioned trade-off between ecosystem preservation and GHG emissions. Recall either Eq (11) or Eq (14) to understand the link between the conceptual framework and this empirical strategy. The first stage of the IV estimation is

$$\Delta FossFuelCap_{c}=\gamma_{0}+\gamma_{1}HydroNotDev_{c}+\gamma_{2}HydroNotDev_{c}^{2}+\gamma_{3}\Delta Emp_{c}+\gamma_{4}\Delta PCInc_{c}+\eta_{d}+v_{c}.$$ (19)

As usual, in the second stage the observed Δ*FossFuelCap* in Eq (18) is replaced with the fitted value of Δ*FossFuelCap* from Eq (19) to obtain the IV estimate for *β*_*1*_. Notice that this estimate is the local average treatment effect (LATE–[14]) of changes in fossil-fuel electricity-generating capacity associated with hydropower not developed because of environmental constraints. That is, the IV estimate for *β*_*1*_ is the average treatment effect for the subset of counties whose fossil-fuel electricity-generating capacity was influenced by the hydropower development regulations.

A major threat to identification is a potential direct effect of hydropower development on GHG emissions. Some studies have shown that while hydroelectric energy is renewable, reservoirs may release carbon dioxide, methane, and nitrous oxide to the atmosphere [36–41]. A few studies had suggested that young reservoirs in low latitudes might produce the largest emissions [42–43], which would mean that most reservoirs in the United States would not be emitting large amounts of GHG. More recent and comprehensive evidence [44] challenges those findings, but indicate that the majority of GHG emissions from reservoir water surfaces is due to methane. Fortunately, my main outcome measure is carbon dioxide emissions. Furthermore, as it has been pointed out recently, new reservoirs could reduce those emissions significantly by clearing the vegetation before flooding, as has been the case in several areas around the world [45].

Observe that I use county as the unit of analysis in this study. It is imperative to explain this choice. It is well-known that electric utilities may consider other locations within their service area to build electricity-generating capacity, or even procure electricity out of their service area. California, for instance, imports about a quarter of its electricity on average from neighboring states (data available at eia.gov/todayinenergy/detail.php?id = 30192). Nevertheless, “[h]istorically, siting of electric power facilities has been regulated mainly on the state and local levels, *with local zoning commissions having the greatest influence*” ([11], p. xxv, my emphasis). “[Z]oning at the local level (…) was endorsed by the U.S. Supreme Court as a legitimate exercise of the police power in 1926, when the Court upheld the validity of a statutory scheme of zoning districts (including zones for industrial uses)” ([30], p.75). “Municipalities can (…) affect the location of energy facilities through local ordinances, land-use planning, and taxation, and taxation policies” ([31], p. 145).

In fact, since the 1970s electric utilities have considered particular counties to site new power plants because of environmental and/or institutional constraints. It has been pointed out that “*[d]ifficulty in licensing sites due to environmental constraints* as well as technological advances in developing acceptably reliable performance from large sites with accompanying economies of scale are promoting the continuance of this trend [to larger site sizes]” ([11], p. xxxix, my emphasis). In particular, “[t]he specific environmental requirements regarding air pollution control that stem from the mandates of the Clean Air Act of 1970 impose a unique set of constraints. For example, it has become infeasible to locate generating facilities near downtown business centers to minimize transmission distances because of present high costs of control equipment and/or the clean fuel necessary for compliance. As a result, downtown sites are being retired and new sites are being located farther away where less stringent controls are required” ([11], p. 2). The “Not In My Back Yard” (NIMBY) activism has also made site selection more difficult, making it a local issue. The *Project No Project*, an initiative of the U.S. Chamber of Commerce that assessed the potential economic impact of permitting challenges facing energy projects, provided evidence that NIMBY activism blocked proposed power plants by organizing local opposition, changing zoning laws, opposing permits, filing lawsuits, and using other long delay mechanisms, “effectively bleeding projects dry of their financing” [12].

In addition, the trade-off between electricity-generating technologies does seem to be salient at the local level. As explained by [11], “[s]everal siting laws require that an electric utility consider alternative sites *or means of generation* when applying for site certification” (p. xxxiii, my emphasis). Indeed, there has been an effort to systematize the comparison across electricity-generating technologies at the county level [13]. It is important to point out, however, that technical considerations might not be the most relevant factor in site/technology selection. As underscored by [11], “[c]orporate policy is perhaps the most powerful, yet most unassessable portion of system planning. It is the prevailing determinant in making decisions among equally viable options or choosing one option despite technical evaluations that might indicate the choice of another” (p. xxii). In fact, as discussed in the energy transition outlook by *Nature Energy* “[d]ecisions about whether or where to build power projects have always been influenced by a complex web of factors in which technical and geographic considerations meet cultural, commercial and political pressures to create the abstract mosaic of power plants that dot the world” ([46], p. S150). In the end, the “[c]osts of building and operating an identical plant across different geographies [counties] will be different” ([13], p. 491).

To emphasize the role of local governments in power plant site selection, I have also reviewed the empirical evidence from the siting of large industrial plants, which may provide useful guidance due to important similarities. Increasingly, local governments compete for new plants by offering substantial subsidies to locate within their jurisdictions [47–48]. Using a quasi-experiment in which counties where new plants choose to locate (i.e., the ‘winners’) are considered treatment counties, and runner-up counties (i.e., the ‘losers’) are considered control counties, those authors have shown that a plant opening increases labor earnings in the new plant’s industry and productivity in incumbent plants in winning counties (relative to losing ones) after the opening of the plant (relative to the period before the opening), without any deterioration in local governments' financial position. Given the similarities between large manufacturing plants and large power plants, and the ubiquity of county competition for new large plants, it is very likely that this analysis serve as guidance for site selection issues faced by electric utilities as well. In fact, “taxing policies of the State and local governments have considerable influence on the economics of building a generating plant in one location as compared to another” ([28], p. 10).

For all these reasons, I use county as the unit of analysis in this study. My conceptual framework and empirical exercise should be seen as an approximation for a decision-making process that includes both permitting and energy planning. Nevertheless, I also report results using electric utility as the unit of analysis in the Results and Discussion section below. Not surprisingly, aggregation at the electric utility level make point estimates larger–there might be more substitution across counties within a service area–but also larger standard errors–there might be more heterogeneity in dealing with those trade-offs across electric utilities.

## Results and discussion

**Fig 1:**
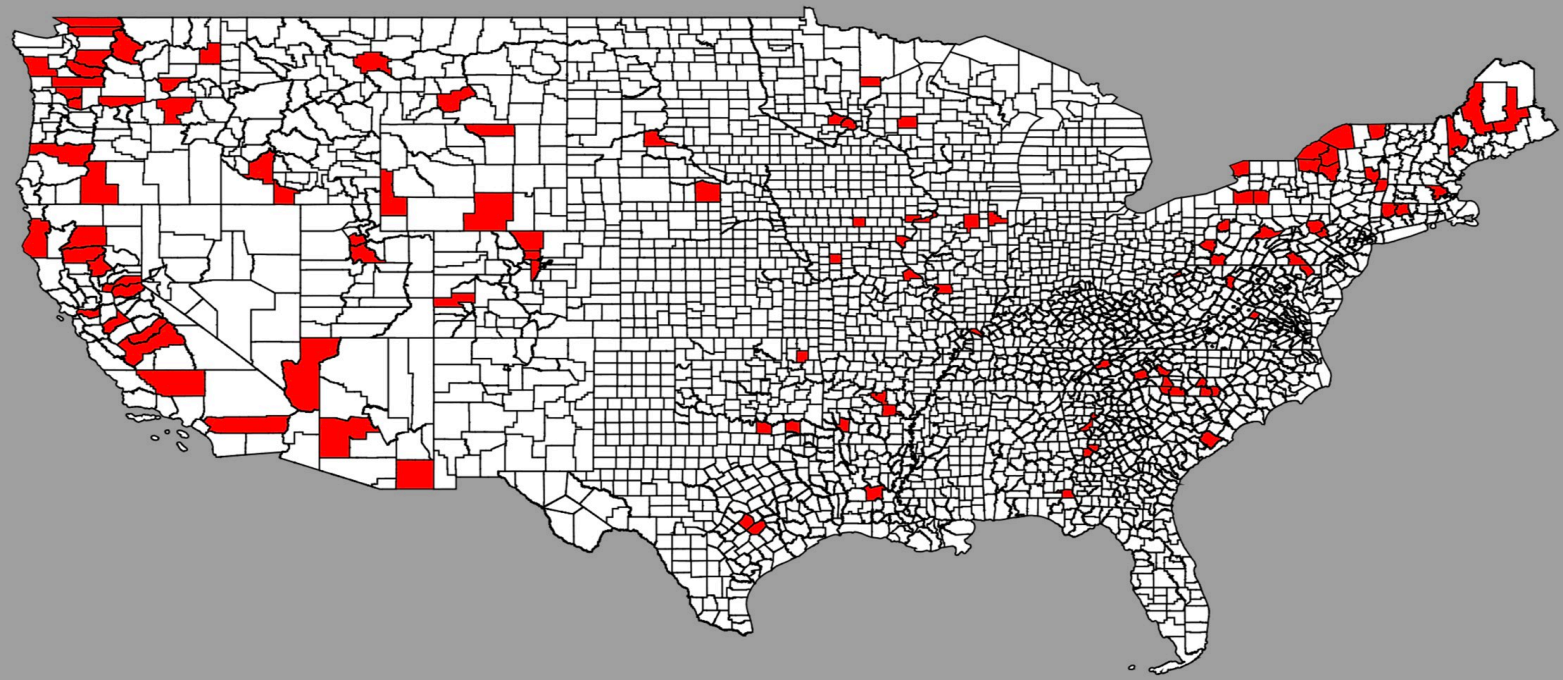
Map of the U.S. counties in the sample. *Notes*: This map shows the counties in the sample for the main empirical analysis. These are counties with at least 30 megawatts of hydropower potential, and that have developed any power plants in the period of analysis– 1998–2014. The sample includes 110 U.S. counties, in 33 states.

The trade-off between ecosystem preservation and GHG emissions is estimated through an instrumental variable approach. The period of analysis is 1998–2014 because the final DOE’s hydropower assessment report was released in 1998, and the latest eGRID emissions data is for 2014. The sample is restricted to counties with at least 30 megawatts of hydropower potential (between the median of 10 megawatts and the 75 percentile of 40 megawatts–results are not sensitive to this choice, as will be discussed below), and that have developed any power plants in the period of analysis. The sample includes 110 U.S. counties, in 33 states, as depicted in Fig 1.

Results are presented in Table 2. Initially, Eq (18) is estimated by OLS. Then, IV estimates are obtained by using potential hydropower not developed due to environmental regulations as a plausibly exogenous shifter in thermal power capacity built in the period of study. The OLS estimate indicates that each new megawatt installed in fossil-fuel power plants over the period 1998–2014 generates, on average, 465 tons of carbon dioxide emissions annually. This suggests that the new large power plants might be driven by a mix of natural gas, coal, and other fossil fuels. S1 Table reports average emissions by each motive power, and shows that new coal plants emit over four times more carbon dioxide than natural gas plants. Other fuels have a large heterogeneity regarding emissions. The preferred IV estimate (Table 2, column 3) reveals that counties with hydropower potential not exploited because of environmental constraints to hydropower development emit three times more carbon dioxide per megawatt (approximately 1,408 tons per megawatt annually, S.E. 563) than suggested by the OLS estimate. (For comparison, the IV estimate is 2,420 tons, S.E. 2,294, when running the analysis at the electric utility level. Therefore, the estimate is not statistically significant. As explained above, aggregation leads to larger and noisier estimates.) Because coal plants emit an average of 2,290 tons of carbon dioxide per megawatt annually, and natural gas 513 (see S1 Table), those environmental regulations seem to induce mixed choices in electricity generation. For reference, the average annual emissions per megawatt of the 2016 U.S. power plant fleet is 1,795 tons (calculations based on data available at eia.gov/electricity/state/unitedstates/). As explained in the empirical strategy in the Materials and Methods section, this increase in the IV estimate suggests a negative bias in the OLS estimate. It is likely that while preferences for environmental amenities may be negatively related to investments in fossil fuel electricity generating capacity, emission levels might be higher around metropolitan areas where economic activity and more-educated, amenity-oriented individuals are more abundant [34–35]. Hence, the negative bias.

**Table 2:** The Impact of fossil fuel electricity generating capacity on carbon dioxide emissions and first stage.

DepVar: ΔCO2 Emissions	OLS	First Stage	IV: 2SLS	IV: LIML	IV: GMM
	(1)	(2)	(3)	(4)	(5)
ΔFossil Fuel Capacity	465.24**		1,408.01**	1,408.74**	1,433.49***
	(199.81)		(562.83)	(563.37)	(305.99)
*Instrumental Variables*					
HydroNotDev		-1.45**			
		(0.68)			
(HydroNotDev^2)/1000		1.67***			
		(0.45)			
*Control Variables*					
ΔTotal Employment	4.88	0.02***	-10.94	-10.95	-11.39***
	(3.37)	(0.00)	(9.16)	(9.17)	(3.56)
ΔPer Capita Income	19.98	-0.01	33.77	33.78	33.54*
	(19.62)	(0.01)	(20.54)	(20.54)	(20.09)
Census Division FE	Yes	Yes	Yes	Yes	Yes
Number of Counties	110	110	110	110	110
R^2	0.34	0.56			
Kleibergen-Paap rk Wald F-statistic			9.353	9.353	9.353
Hansen J-statistic			0.00285	0.00285	0.00279
P-value J-statistic			0.957	0.957	0.958

*Notes*: This table reports the results of regressions of changes in annual carbon dioxide emissions over 1998–2014 on changes in fossil fuel electricity generating capacity over the same period. The estimating specification is Eq (18) in the Materials and Methods section. Column 1 presents the OLS estimates, and columns 3–5 the instrumental variable (IV) estimates using two stage least squares (2SLS), limited-information maximum likelihood (LIML), known to be less precise but also less biased than IV, and by continuously-updated GMM, known to perform better than two-step feasible GMM in small samples. Column 2 presents the first stage OLS regression of changes in fossil fuel electricity generating capacity over 1998–2014 on a quadratic function on hydropower potential not developed because of ecosystem preservation regulations. For clarity, the Kleibergen-Paap rk Wald F-statistic is a test statistic for a test of weak instruments. “Weak identification” arises when the instruments are correlated with the endogenous regressors, but only weakly. Furthermore, the Hansen’s J-statistic is a test statistic for a test of overidentifying restrictions. The joint null hypothesis is that the instruments are valid, i.e., uncorrelated with the error term, and that the excluded instruments are correctly excluded from the estimated equation. A rejection would cast doubt on the validity of the instruments. Standard errors clustered at the state level are reported in parentheses.

*** represents statistically significant at 1 percent level

** at 5 percent

* at 10 percent.

The first stage regression also brings insights on the impact of environmental regulations on the energy mix. It sheds some light on how restricting construction of hydroelectric dams affects the development of new thermal power plants. When I use the number of megawatts of hydropower that cannot be developed because of environmental constraints (*HydroNotDev*) and its square as instruments, both coefficients are statistically significant, and the Kleibergen-Paap rk Wald F-statistic is about 9.35, close to the rule-of-thumb of 10 for relatively strong first stage. For clarity, the Kleibergen-Paap rk Wald F-statistic is a test statistic for a test of weak instruments. “Weak identification” arises when the instruments are correlated with the endogenous regressors, but only weakly. Furthermore, the Hansen’s J-test for overidentifying restrictions reveals that the model is “valid”. Recall that the joint null hypothesis is that the instruments are valid, i.e., uncorrelated with the error term, and that the excluded instruments are correctly excluded from the estimated equation. A rejection would cast doubt on the validity of the instruments, which does not seem the case in this setting.

**Fig 2:**
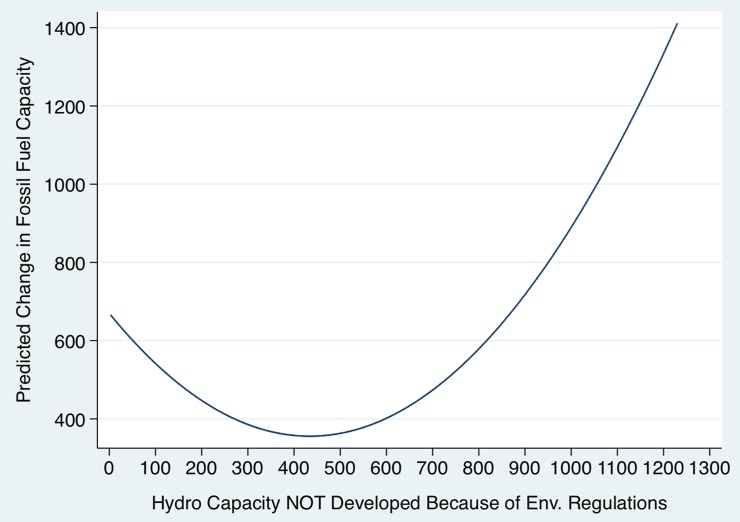
Predicted change in fossil fuel electricity generating capacity: 1998–2014. *Notes*: This figure plots the first stage relationship between change in fossil fuel electricity generating capacity and hydropower potential not developed because of the ecosystem preservation regulations. This convex relationship was estimated by Eq (19) of the Materials and Methods section. The range considered in the x-axis is the range observed in the data. See S6 Table for a list of the twenty counties in the sample with the highest values of hydro capacity not developed because of ecosystem preservation regulations.

Table 2 (column 2) reports the first stage coefficients, and reveal a convex relationship between *HydroNotDev* and Δ*ThermDev* with a minimum at 434 megawatts, as depicted in Fig 2. This figure shows that, for low levels of *HydroNotDev*, additional environmental constraints are associated with decreases in the number of megawatts developed in fossil-fuel power plants. This may be due to either stricter environmental regulations to start with, or low hydropower potential. Since fossil fuel power plants also need authorization to operate (e.g., due to air pollution constraints associated with the Clean Air Act, as mentioned in Materials and Methods section), and also require large volumes of water for cooling, those locations might be undesirable for both types of power plants. However, for higher levels of *HydroNotDev*, a positive relationship seems to emerge. One possible explanation is that, after a certain threshold, restrictions imposed by hydroelectric licensing rules may be used as leverage by electric utilities to obtain permits to build new thermal power plants. Indeed, as pointed out by [30], “[a] selection of concerns which are frequently raised in siting proceedings may be readily identified. Competition for water supplies between electric utilities and other industrial, municipal and agricultural users is complicated by instream flow needs for fish and wildlife, for recreational uses and for nonpoint source pollution dilution. This competition is often intense” ([30], p. 77). Recall that “[w]ater in sufficient quantities and with low enough temperature must be available to absorb the maximum plant heat release without raising the temperature of the receiving waters above satisfactory levels” ([28], p. 16).

In addition, as explained previously, one of the biggest challenges in siting new plants is resistance from local communities. Citizen groups argue that power plants are a source of numerous negative local externalities, including visual disamenities and noise. [49] provides empirical evidence suggesting that such claims might be well-founded. Examining housing values and rents for neighborhoods in the U.S. where power plants were opened during the 1990s, he finds that neighborhoods within 2 miles of plants experienced 3 to 7 percent decreases in housing values and rents, with some evidence of larger decreases within 1 mile and for large-capacity plants. Although impacts on the housing market are often assumed to provide a summary measure for the net change in welfare [50]–the costs and benefits of attracting a power plant should be capitalized into the price of land/housing units–it is imperative to highlight other effects of fossil fuel power plants on outcomes of societal interest such as fertility and infant health [51–54].

It is important to mention that I did not find statistically significant effects on emissions of local pollutants–sulfur dioxide and nitrogen oxides–as reported in S2 Table. Also, the impact on a measure of carbon dioxide equivalent, which includes two other GHG (methane and nitrous oxide) and is available from 2005 onwards, was also imprecisely estimated, but we cannot rule out that it was of similar magnitude as the main effect on carbon dioxide reported above.

### Weak identification

There may be some weak identification issues in the main analysis. The F- statistic for the first stage with instruments *HydroNotDev* and its square is slightly below the “safe threshold” of 10 suggested by [55]. When instruments are only weakly correlated with the endogenous explanatory variable, the IV estimates might be biased towards the OLS coefficients. For this reason, I also estimated the model by limited-information maximum likelihood (LIML), known to be less precise but also less biased than IV, and by continuously-updated generalized method of moments (GMM), known to perform better than two-step feasible GMM in small samples. As shown in Table 2 (columns 4 and 5), in both cases the coefficient of interest increases only slightly, suggesting that the bias in the IV estimates might be negligible.

### Robustness checks

I have conducted sensitivity analysis for two important issues regarding alternative samples and specifications. First, I have rerun the analysis using main specification (Eq 18), but allowing the sample to include counties with at least 10 megawatts of hydropower potential instead of 30 megawatts in the main analysis, or restricting the sample to include only counties with hydropower potential of 40 megawatts or more. Recall that the overall median of hydropower potential is 10 megawatts, and the 75 percentile 40 megawatts. Because these alternative samples may change the composition of counties affected by the instruments, one would expect changes in the IV estimate for *β*_*1*_. Recall the LATE interpretation of this estimate: it is the average treatment effect for the counties whose fossil-fuel electricity-generating capacity was influenced by the environmental constraints on hydropower development. As reported in S3 Table, the IV estimates for these alternative samples are remarkably similar to the main estimates, indicating that the findings of this study might be relatively general regarding the trade-off between environmental regulations restricting hydropower development and GHG emissions.

Second, I have considered the role of accessibility in siting decisions. In fact, besides “proximity of a direct water source”, “[t]he decision about the location of a power plant is dictated by proximity to load centers, land requirements, fuel supply and *transportation access*” ([29], p. 506, my emphasis). Because the other siting criteria are implicitly controlled for in Eq (18), as explained in the empirical strategy, and accessibility may also determine GHG emissions, in this robustness check I have included only two additional controls to address concerns regarding changes in access to transportation: an indicator for whether a county was supposed to receive a portion of the interstate highway system based on the initial plan released in 1944 [56–57], and the mileage of railroads in each county by 1911 [58]. In 1941, President Roosevelt appointed a National Interregional Highway Committee. This committee was headed by the Commissioner of Public Roads, and appears to have been professional, rather than political ([57]). The highways were designed to address three policy goals ([57]). First, they intended to improve the connection between major metropolitan areas in the U.S. Second, they were planned to serve U.S. national defense. And finally, they were designed to connect with major routes in Canada and Mexico. Congress acted on these recommendations in the Federal-Aid Highway Act of 1944. In my analysis, I refer to the plan recommended by that committee as the “1944 plan”. The construction of the Interstate Highway System began after funding was approved in 1956, and by 1975 the system was mostly complete, spanning over 40,000 miles. Regarding the railroads data, I have used historical data for 1911 because this was the last available in [58]. This is without loss of generality. The idea is to weaken the association between local preferences for GHG emissions across generations.

Recall that because Eq (18) is differenced, including those indicators translates into allowing for differential time trends based on those two baseline measures of accessibility. I exploit variation arising from the highway plan instead of the actual construction of interstate highways to avoid the endogeneity related to local preferences for GHG emissions. Indeed, the local willingness to invest in transportation infrastructure might be associated with the willingness to invest in energy infrastructure projects that might be more intensive in GHG emissions. In fact, because politicians pushed for changes in highway routes in response to economic and demographic conditions of their constituencies [56–57], other local infrastructure projects might have been affected as well. Similarly, I use the mileage of railroads more than a century ago to weaken, potentially break, the association between local preferences for GHG emissions across generations. As reported in S4 Table, the IV estimate for *β*_*1*_ is again remarkably similar to the main estimate, suggesting that the main specification is indeed a parsimonious model to examine the trade-off considered in this study.

### Falsification test

Because there are many environmental regulations that are unrelated to habitat preservation, such as land for historical and cultural monuments, as reported in the Materials and Methods section, I am able to run a falsification test using them as the underlying force in the first stage. As S5 Table reveals, the coefficients in the first stage are similar in magnitude as in the first stage for the main analysis, but both coefficients are imprecisely estimated. The IV estimate, however, is very different from the main finding. If anything, it suggests a negative effect on GHG emissions in those counties with large undeveloped hydropower potential. Given that historical and cultural monuments, for example, are more visible to the public, it might be that those potential dam sites are not used by electric utilities for bargaining in obtaining permits for the construction of fossil fuel power plants. This result reinforces the main findings that ecosystem preservation regulations are the driving force behind the substitution between hydro and fossil fuel power plants.

## Conclusions

Do environmental regulations aimed at preserving natural ecosystems protect the environment? The answer seems to be not necessarily. Here we presented evidence that, while hydroelectric licensing rules preserved the wilderness and wildlife by restricting the development of hydroelectric projects, they may have led to more GHG emissions. Basically, the ecosystem preservation regulations gave rise to a replacement of hydropower, which is a renewable, relatively low-emitting source of energy, with conventional fossil-fuel power, which is highly polluting. Restrictions imposed by hydroelectric licensing rules might be used as leverage by electric utilities to obtain permits to expand thermal power generation. Each megawatt of fossil fuel power-generating capacity added to the power grid because of environmental constraints on hydropower development led to an increase in annual carbon dioxide emissions of about 1,400 tons, close to the average annual emissions per megawatt of the 2016 U.S. power plant fleet– 1,795 tons. Environmental regulations focusing only on the preservation of ecosystems appear to have encouraged electric utilities to substitute dirtier fuels for electricity generation.

As pointed out before, these findings highlight the pernicious incentives of incomplete environmental regulations, and points to the importance of an integrated regulatory framework that includes both ecosystem preservation and GHG emissions. If the government seeks to preserve nature, it may have to simultaneously restrict land use and emissions. A similar regulatory framework may be useful to guide the debate on the development of other renewable energy sources, such as wind and solar energy. Also, the empirical evidence of unintended consequences of land ecosystem preservation regulations provides guidance for a more balanced cost-benefit analysis of hydroelectric dams. The Three Gorges Dam in China, the world’s largest hydroelectric project, for example, has raised international concerns about environmental damages, but few organizations recognize the sizeable amount of low-carbon electric power generated [59]. Also, the historical involvement of the World Bank with the construction of large hydro dams in Asia, Africa and Latin America has been criticized by environmentalists. If some of those projects were not executed due to ecosystem preservation concerns, it is possible that the unintended consequences may have been GHG emissions. Again, bidimensional negotiations, integrating both ecosystem preservation and emissions concerns, might have been more effective in protecting the environment. Moving forward, jurisdictions could, for instance, impose both environmental regulations to protect habitat and reduce GHG emissions.

## Supporting information

S1 Table The Impact of fossil fuel electricity generating capacity on carbon dioxide emissions by fossil fuel. *Notes*: This table reports the results of OLS regressions of changes in annual carbon dioxide emissions over 1998–2014 on changes in fossil fuel electricity generating capacity over the same period by fossil fuel. The estimating specification is Eq (18) in the Materials and Methods section. Standard errors clustered at the state level are reported in parentheses. *** represents statistically significant at 1 percent level, ** at 5 percent, and * at 10 percent.
(PNG)

S2 Table The Impact of fossil fuel electricity generating capacity on air emissions of other pollutants and first stage. *Notes*: This table reports the results of regressions of changes in annual air emissions of sulfur dioxide, nitrogen oxides, and carbon dioxide equivalent (including methane and nitrous oxide) over 1998–2014 on changes in fossil fuel electricity generating capacity over the same period. The table replicates Table 2 (columns 1–3) for each additional pollutant. Standard errors clustered at the state level are reported in parentheses. *** represents statistically significant at 1 percent level, ** at 5 percent, and * at 10 percent.
(PNG)

S3 Table The Impact of fossil fuel electricity generating capacity on carbon dioxide emissions and first stage for alternative samples–robustness Check 1. *Notes*: This table replicates the results of regressions of changes in annual carbon dioxide emissions over 1998–2014 on changes in fossil fuel electricity generating capacity over the same period reported in Table 2 (columns 1–3) with alternative samples. Columns 1–3 use 201 counties with hydropower potential above 10 megawatts, and columns 4–6 use 96 counties with hydropower potential above 40 megawatts. Standard errors clustered at the state level are reported in parentheses. *** represents statistically significant at 1 percent level, ** at 5 percent, and * at 10 percent.
(PNG)

S4 Table The Impact of fossil fuel electricity generating capacity on carbon dioxide emissions and first stage with controls for accessibility–robustness Check 2. *Notes*: This table reports results of regressions of changes in annual carbon dioxide emissions over 1998–2014 on changes in fossil fuel electricity generating capacity over the same period reported in Table 2 (columns 1–3), but adding controls for accessibility–an indicator for whether a county was supposed to receive a highway as recommended by the 1944 Interstate Highway System plan, and the 1911 mileage of railroads within a county. Standard errors clustered at the state level are reported in parentheses. *** represents statistically significant at 1 percent level, ** at 5 percent, and * at 10 percent.
(PNG)

S5 Table The impact of fossil fuel electricity generating capacity on carbon dioxide emissions using alternative land regulations–falsification test. *Notes*: This table replicates the results of regressions of changes in annual carbon dioxide emissions over 1998–2014 on changes in fossil fuel electricity generating capacity over the same period reported in Table 2 (columns 1–3) with alternative land regulations such as land for historical and cultural monuments, and for scenic and geologic value. It is a falsification test in the sense that they may not be used for electric utilities in bargaining with FERC to obtain permits to site new fossil fuel power plants because there is not trade-off between hydropower and fossil fuels in electricity generation. Standard errors clustered at the state level are reported in parentheses. *** represents statistically significant at 1 percent level, ** at 5 percent, and * at 10 percent.
(PNG)

S6 Table List of twenty counties with the highest values of *HydroNotDev* in the sample. *Notes*: This tables reports the list of twenty counties in the sample with the highest values of *HydroNotDev*, as well as the fossil fuel electricity generating capacity developed in those counties.
(PNG)
