# Supplementary figures and images for: The unintended impact of ecosystem preservation on greenhouse gas emissions: Evidence from environmental constraints on hydropower development in the United States
Source: PLoS One. 2019 Jan 10;14(1):e0210483. doi: 10.1371/journal.pone.0210483 (PMC6328135; doi:10.1371/journal.pone.0210483)

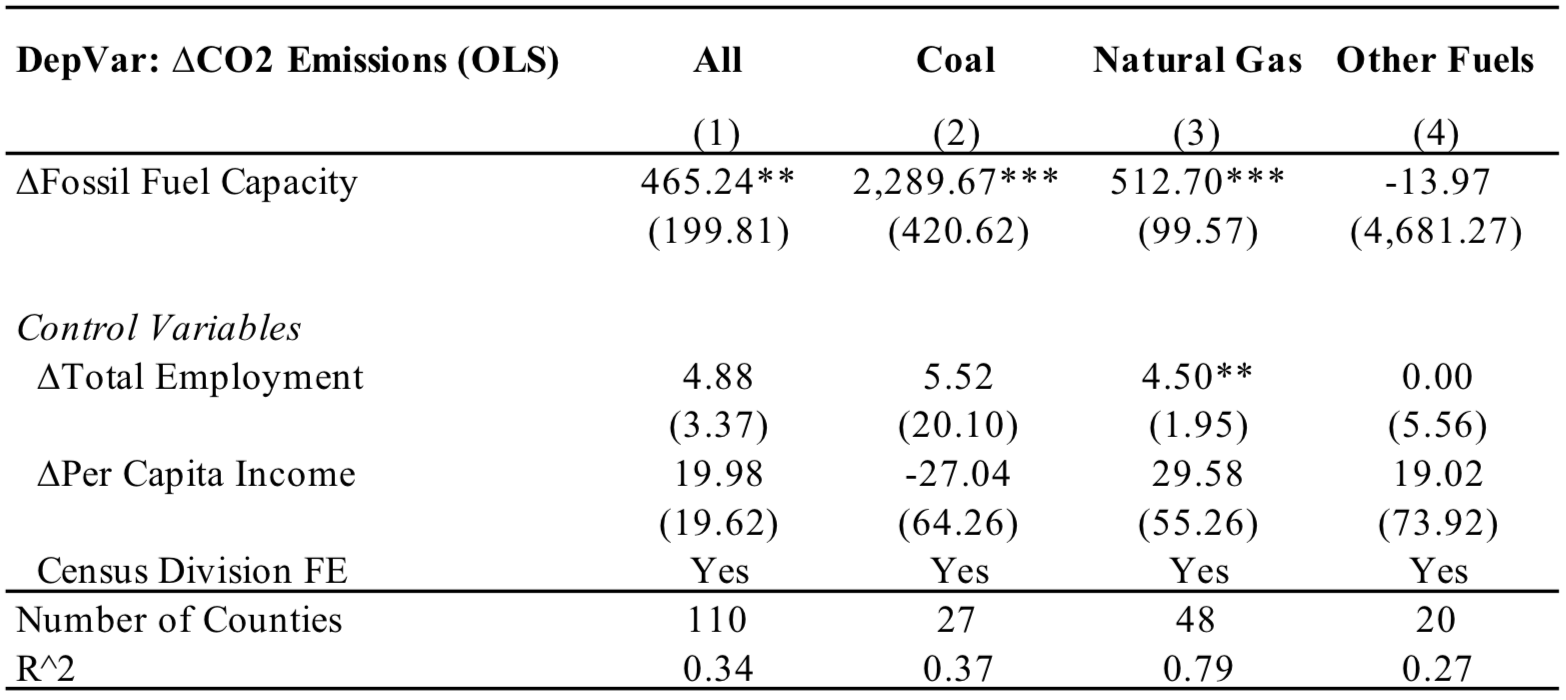

Supplement: S1 Table — Notes: This table reports the results of OLS regressions of changes in annual carbon dioxide emissions over 1998–2014 on changes in fossil fuel electricity generating capacity over the same period by fossil fuel. The estimating specification is Eq (18) in the Materials and Methods section. Standard errors clustered at the state level are reported in parentheses. *** represents statistically significant at 1 percent level, ** at 5 percent, and * at 10 percent. (PNG) [file pone.0210483.s001.png]

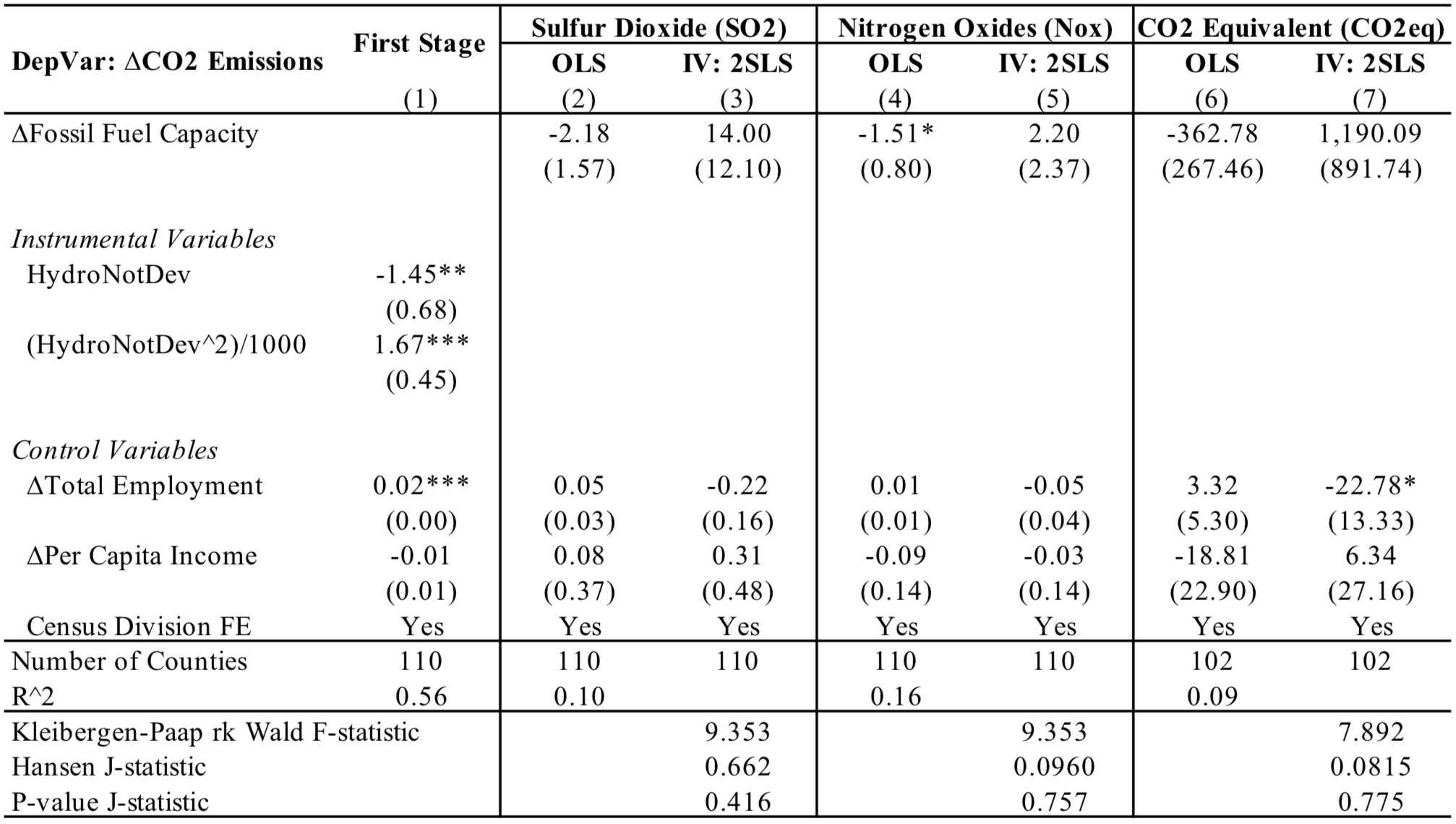

Supplement: S2 Table — Notes: This table reports the results of regressions of changes in annual air emissions of sulfur dioxide, nitrogen oxides, and carbon dioxide equivalent (including methane and nitrous oxide) over 1998–2014 on changes in fossil fuel electricity generating capacity over the same period. The table replicates Table 2 (columns 1–3) for each additional pollutant. Standard errors clustered at the state level are reported in parentheses. *** represents statistically significant at 1 percent level, ** at 5 percent, and * at 10 percent. (PNG) [file pone.0210483.s002.png]

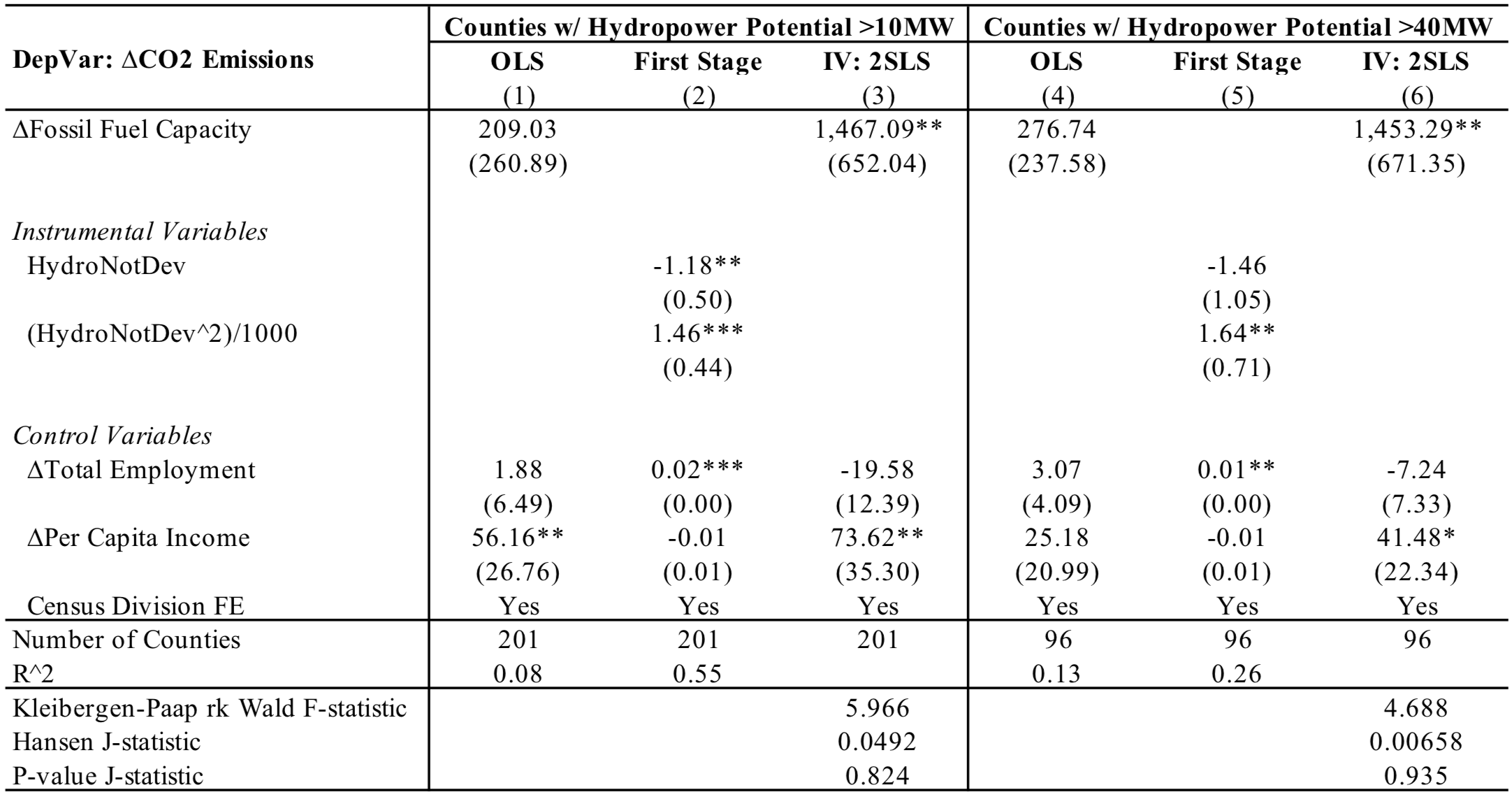

Supplement: S3 Table — Notes: This table replicates the results of regressions of changes in annual carbon dioxide emissions over 1998–2014 on changes in fossil fuel electricity generating capacity over the same period reported in Table 2 (columns 1–3) with alternative samples. Columns 1–3 use 201 counties with hydropower potential above 10 megawatts, and columns 4–6 use 96 counties with hydropower potential above 40 megawatts. Standard errors clustered at the state level are reported in parentheses. *** represents statistically significant at 1 percent level, ** at 5 percent, and * at 10 percent. (PNG) [file pone.0210483.s003.png]

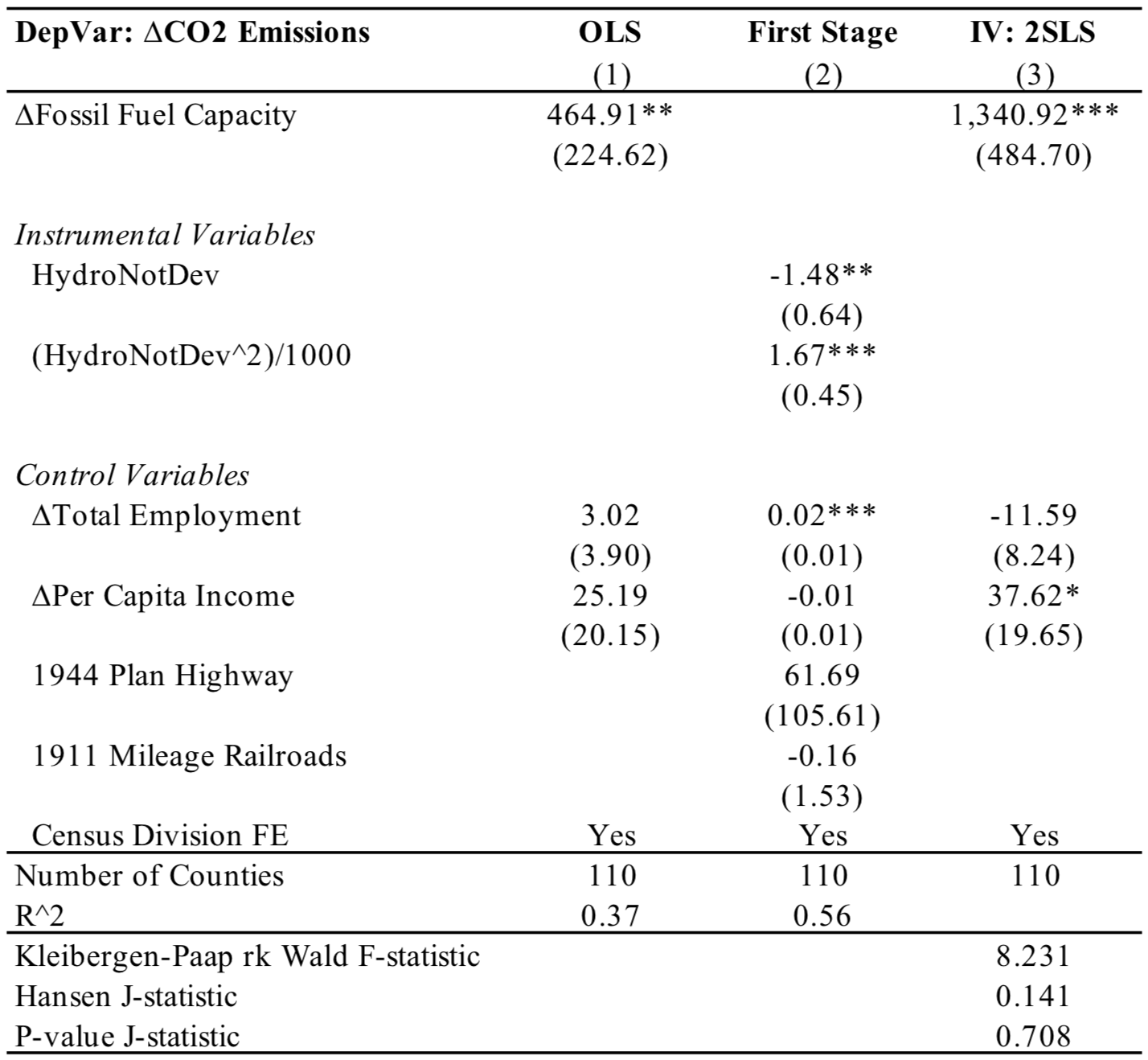

Supplement: S4 Table — Notes: This table reports results of regressions of changes in annual carbon dioxide emissions over 1998–2014 on changes in fossil fuel electricity generating capacity over the same period reported in Table 2 (columns 1–3), but adding controls for accessibility–an indicator for whether a county was supposed to receive a highway as recommended by the 1944 Interstate Highway System plan, and the 1911 mileage of railroads within a county. Standard errors clustered at the state level are reported in parentheses. *** represents statistically significant at 1 percent level, ** at 5 percent, and * at 10 percent. (PNG) [file pone.0210483.s004.png]

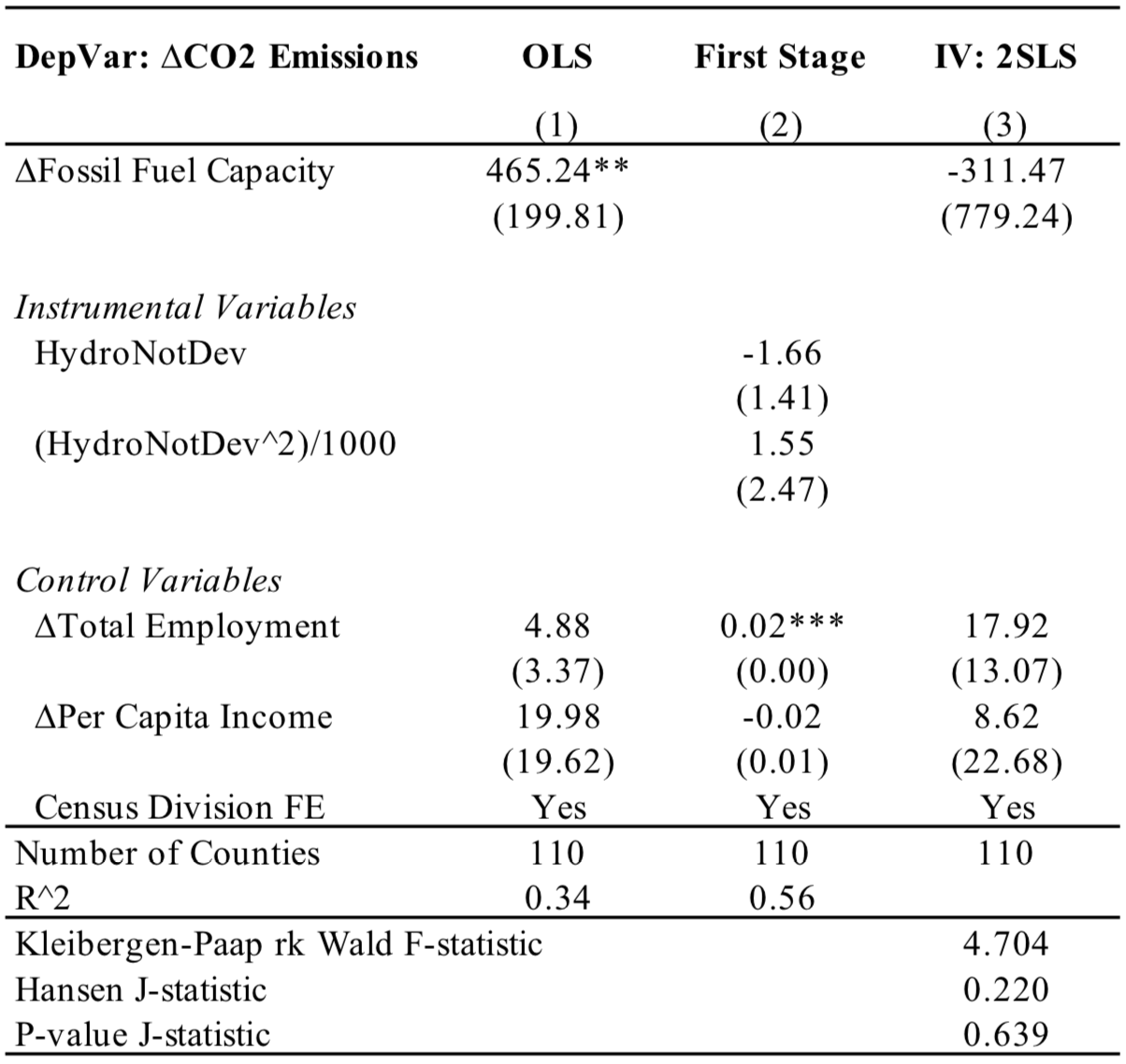

Supplement: S5 Table — Notes: This table replicates the results of regressions of changes in annual carbon dioxide emissions over 1998–2014 on changes in fossil fuel electricity generating capacity over the same period reported in Table 2 (columns 1–3) with alternative land regulations such as land for historical and cultural monuments, and for scenic and geologic value. It is a falsification test in the sense that they may not be used for electric utilities in bargaining with FERC to obtain permits to site new fossil fuel power plants because there is not trade-off between hydropower and fossil fuels in electricity generation. Standard errors clustered at the state level are reported in parentheses. *** represents statistically significant at 1 percent level, ** at 5 percent, and * at 10 percent. (PNG) [file pone.0210483.s005.png]

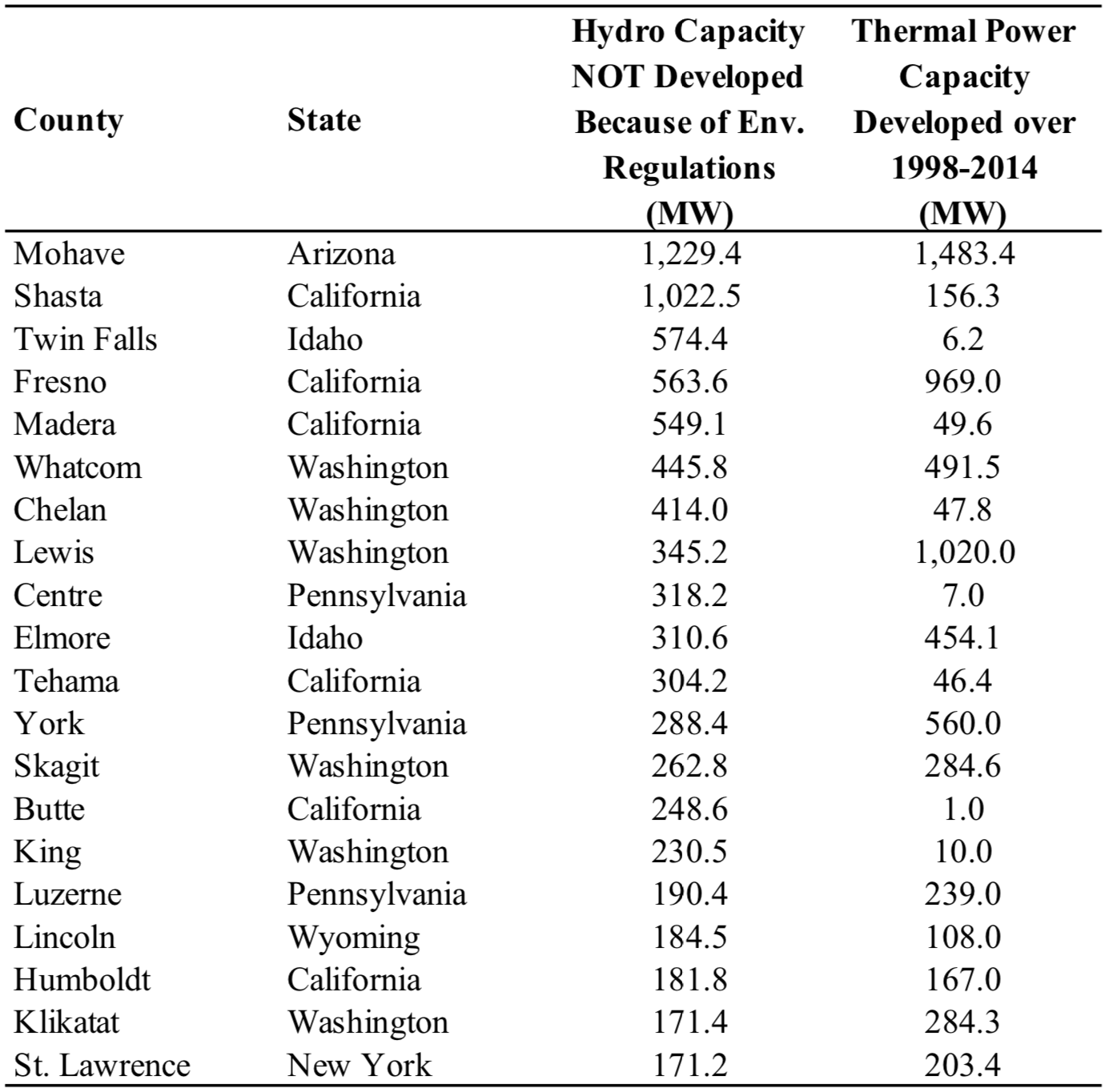

Supplement: S6 Table — Notes: This tables reports the list of twenty counties in the sample with the highest values of HydroNotDev, as well as the fossil fuel electricity generating capacity developed in those counties. (PNG) [file pone.0210483.s006.png]
